# Genomic loss in environmental and isogenic morphotype isolates of *Burkholderia pseudomallei* is associated with intracellular survival and plaque-forming efficiency

**DOI:** 10.1371/journal.pntd.0008590

**Published:** 2020-09-29

**Authors:** Natnaree Saiprom, Tanes Sangsri, Sarunporn Tandhavanant, Sineenart Sengyee, Rungnapa Phunpang, Anucha Preechanukul, Uriwan Surin, Apichai Tuanyok, Ganjana Lertmemongkolchai, Wasun Chantratita, T. Eoin West, Narisara Chantratita

**Affiliations:** 1 Department of Microbiology and Immunology, Faculty of Tropical Medicine, Mahidol University, Bangkok, Thailand; 2 Department of Microbiology, Princess of Naradhiwas University, Narathiwat, Thailand; 3 Mahidol-Oxford Tropical Medicine Research Unit, Faculty of Tropical Medicine, Mahidol University, Bangkok, Thailand; 4 Department of Medical Laboratory, Nakhon Phanom Hospital, Nakhon Phanom, Thailand; 5 Department of Infectious Diseases and Immunology, College of Veterinary Medicine, University of Florida, Gainesville, FL, United States of America; 6 Centre for Research and Development of Medical Diagnostic Laboratories, Department of Clinical Immunology, Faculty of Associated Medical Science, Khon Kaen University, Khon Kaen, Thailand; 7 Center for Medical Genomics, Faculty of Medicine Ramathibodi Hospital, Mahidol University, Bangkok, Thailand; 8 Division of Pulmonary, Critical Care & Sleep Medicine, Harborview Medical Center, University of Washington, Seattle, WA, United States of America; University of Texas Medical Branch, UNITED STATES

## Abstract

**Background:**

*Burkholderia pseudomallei* is an environmental bacterium that causes melioidosis. A facultative intracellular pathogen, *B*. *pseudomallei* can induce multinucleated giant cells (MNGCs) leading to plaque formation *in vitro*. *B*. *pseudomallei* can switch colony morphotypes under stress conditions. In addition, different isolates have been reported to have varying virulence *in vivo*, but genomic evolution and the relationship with plaque formation is poorly understood.

**Methodology/Principle findings:**

To gain insights into genetic underpinnings of virulence of *B*. *pseudomallei*, we screened plaque formation of 52 clinical isolates and 11 environmental isolates as well as 4 isogenic morphotype isolates of *B*. *pseudomallei* strains K96243 (types II and III) and 153 (types II and III) from Thailand in A549 and HeLa cells. All isolates except one environmental strain (A4) and K96243 morphotype II were able to induce plaque formation in both cell lines. Intracellular growth assay and confocal microscopy analyses demonstrated that the two plaque-forming-defective isolates were also impaired in intracellular replication, actin polymerization and MNGC formation in infected cells. Whole genome sequencing analysis and PCR revealed that both isolates had a large genomic loss on the same region in chromosome 2, which included Bim cluster, *T3SS-3* and *T6SS-5* genes.

**Conclusions/Significance:**

Our plaque screening and genomic studies revealed evidence of impairment in plaque formation in environmental isolates of *B*. *pseudomallei* that is associated with large genomic loss of genes important for intracellular multiplication and MNGC formation. These findings suggest that the genomic and phenotypic differences of environmental isolates may be associated with clinical infection.

## Author summary

This study used a plaque-formation screen, as a surrogate for bacterial virulence, to identify a plaque-defective environmental isolate of *B*. *pseudomallei* that is impaired in intracellular replication, actin polymerization and MNGC formation in infected cells. Whole genome sequencing and PCR indicated that this phenotype was attributable to genomic loss. A similar event was detected in a K96243 isogenic morphotype *in vitro* under a laboratory stress condition. In contrast, all isolates from clinical samples induced high plaque-forming efficiency. Our data suggest that further studies are required to identify the distribution of less virulent strains in the environment and the correlation with human melioidosis.

## Introduction

*Burkholderia pseudomallei* is a Gram-negative intracellular bacterium and the causative agent of melioidosis, a severe infectious disease in humans and animals. It is a biothreat Tier 1 select agent but widely spread in the environment in Southeast Asia, particularly Thailand, and Northern Australia [1, 2]. *B*. *pseudomallei* is a saprophytic bacterium with a high genetic diversity [3, 4]. Although clinical isolates of *B*. *pseudomallei* appear to be genetically distinct from environmental strains, the bacterium in the environment is considered the major source of clinical infection [3–5]. Rice farmers and individuals with underlying diseases are considered high-risk groups for infection with *B*. *pseudomallei* especially during the monsoon seasons [6–8]. Infection occurs by inoculation through skin abrasions, ingestion, or inhalation. The clinical features of melioidosis vary considerably, ranging from acute fulminant septicemia to chronic localized infection. In its acute form, death can occur within days of the onset of symptoms. The mortality rate of melioidosis exceeds 40% in Northeast Thailand, and modeling has estimated that 165,000 cases of human melioidosis occur annually worldwide [9]. The disease can be treated with intravenous ceftazidime or meropenem followed by oral trimethoprim–sulfamethoxazole for 3–6 months [10]. Unfortunately, there is currently no licensed vaccine available for prevention. Melioidosis is therefore a major threat to human and animal health.

*B*. *pseudomallei* can persist in the environment for long periods [11]. Persistence in hosts is recognized and relapse occurs in humans [2, 12–14]. *B*. *pseudomallei* can invade various cell types [15, 16]. The intracellular life cycle of *B*. *pseudomallei* is complex and requires various virulence factors. Following invasion, *B*. *pseudomallei* produces enzymes to protect from being killed by reactive oxygen species (ROS) and survives under oxidative stress conditions in endocytic vacuoles [17–19]. It escapes from endocytic vacuoles by a functional type 3 secretion system (T3SS)-3 [20] in which T3SS-3 effectors, BopA and BopC, facilitate escape from the phagolysosome and autophagic vesicles [21–23]. After egress from vesicles, *B*. *pseudomallei* replicates and spreads from cell to cell, inducing multinucleated giant cell (MNGC) formation and eventually plaque formation. The cell-to-cell spread requires intracellular movement. *B*. *pseudomallei* induces actin-tail formation by BimA and BimC that induces F-actin polymerization at the rear pole of the bacteria [24, 25]. The intracellular movement of *B*. *pseudomallei* generates protrusions from infected cells, which facilitates contact with adjacent cells resulting in MNGC formation [20, 26]. This process requires type VI secretion system (T6SS)-5 in which hemolysin-coregulated protein (Hcp-5), a T6SS-5 tube structure and effector molecule, plays an important role [27, 28]. T6SS-5 is positively regulated by BsaN, VirAG and BprC and negatively regulated by quorum sensing system [16, 27, 29, 30]. After multiplication intracellularly, *B*. *pseudomallei* can generate plaques in monolayers *in vitro* as indicated by dead cells centrally surrounded by live infected cells. Defects in MNGC and plaque formation have been shown to be associated with mutation deletions of these virulence genes [20, 23, 25, 27]. The role of these genes has been confirmed in animal models, as the mutants are less virulent [19, 25, 27].

Some genotypes of *B*. *pseudomallei* predominate in clinical isolates but they are uncommon in the environment [3–5]. We previously demonstrated that *B*. *pseudomallei* undergoes colony switching under several stress conditions *in vivo* and *in vitro* [31, 32]. Variation in morphology is known to be associated with changes in the proteome and virulence [31–33]. There is a need to understand the difference in virulence among *B*. *pseudomallei* strains in different collections, but this requires a robust virulence screening method. Plaque formation is a phenotypic characterization technique that is used as a surrogate for virulence of intracellular microbes such as *Rickettsia rickettsii*, *Shigella flexneri*, *Listeria monocytogenes* as well as *B*. *pseudomallei* and *B*. *thailandensis* [34–41]. Recently, plaque formation has been used as a high-throughput screen for inhibitors of the intracellular *B*. *pseudomallei* lifecycle [42].

The increasing application of whole genome sequencing brings a new level of information on relatedness and characterization of the virulence of bacteria [43]. Several studies have used genome sequencing to characterize *B*. *pseudomallei* ecology and genetic diversity in Thailand [44, 45] but has not been performed to define a genetic mechanism of less virulent strains. We hypothesized that combining plaque-forming assays of environmental and clinical *B*. *pseudomallei* isolates with targeted whole genome sequencing of isolates with abnormal phenotypes could identify genetic underpinnings that may be required for virulence. Here, we describe the findings of a plaque-forming screen of *B*. *pseudomallei* isolates from different clinical and environmental sources in Thailand and isogenic colony morphology types. The isolates defective in plaque-forming were further characterized phenotypically for host cell infection, intracellular replication, actin-tail formation, MNGC formation, and characterized genetically by whole genome sequencing and PCR.

## Materials and methods

### Bacterial strains

Sixty-seven *B*. *pseudomallei* isolates were used in this study. These included (i) 50 clinical isolates from melioidosis patients who admitted to Nakhon Phanom Hospital, Nakhon Phanom, Northeast Thailand between October 2015 to November 2016, (ii) K96243 (referred to as K96243 type I) and strain 153 (referred to as 153 type I) from melioidosis patients in Khon Kaen and Ubon Ratchathani, Northeast Thailand, respectively [31, 46], (iii) 11 environmental *B*. *pseudomallei* from soil samples in Ubon Ratchanthani, Northeast Thailand in 2005 [47] and 4 isogenic morphotype isolates of strain K96243 (referred to as K96243 types II and type III) and strain 153 (referred to as 153 types II and III) [31, 32]. The isogenic morphotypes II and III of *B*. *pseudomallei* were generated from nutritional starvation of K96243 and 153 [31, 32]. Colony morphology type I, II and III were confirmed by subculture on Ashdown agar and incubated at 37°C in air for 4 days as previously described [31].

### Ethical approval

The study was approved by the Ethics Committees of the Faculty of Tropical Medicine, Mahidol University (approval number MUTM 2015-002-02) and Nakhon Phanom Hospital, Nakhon Phanom (approval number IEC-NKP1-No.15/2558), Thailand. Written informed consent was obtained from all subjects enrolled in this study. All research was performed in accordance with relevant guidelines and regulations.

### Enrollment

A prospective cohort study of melioidosis patients was conducted at Nakhon Phanom Hospital during October 2015 to November 2016. *B*. *pseudomallei* positive culture results from hospital’s microbiology laboratory were reviewed daily for screening the potential study subjects. Patients who met the criteria of enrollment (age 15 years or older, admitted to hospital, culture positive for *B*. *pseudomallei* within last 24 hours) and provided written informed consent/assent were enrolled. 50 *B*. *pseudomallei* isolates from 49 melioidosis patients were evaluated for plaque formation efficiency. All 49 isolates were obtained on the first day of enrollment but one isolate was obtained from a patient with relapsed infection.

### Bacterial growth curve analysis

One colony of *B*. *pseudomallei* was inoculated in 3 ml of LB. The culture was incubated at 37°C with shaking at 200 rpm for 18 h. Bacteria were collected by centrifugation at 12,000 rpm for 5 min and then washed PBS. Bacterial pellet was suspended in PBS and adjusted the optical density (OD) at 600 nm to obtain a bacterial concentration of approximately 1 × 10^8^ CFU/ml. Ten microliters of bacterial suspension was added to 10 ml of LB broth to make a final concentration of 1 × 10^5^ CFU/ml. Cultures were incubated 37°C with shaking at 200 rpm. The viable count was performed by sampling 100 μl of culture at time intervals (0-, 2-, 4-, 6-, 12-, 24 h), diluted in PBS and inoculated on Columbia agar in triplicate. The plates were incubated at 37°C in air for 16 h. These investigations were performed in two independent experiments.

### Cell lines and culture conditions

Two cell lines were used in this study. HeLa cells (human cervix carcinoma epithelium cells) were maintained in Dulbecco’s Modified Eagle’s medium (DMEM, Invitrogen). A549 cells (human lung epithelial cells) were maintained in Roswell Park Memorial Institute (RPMI) 1640 medium (Invitrogen). Cell culture medium was supplemented with 10% heat-inactivated fetal bovine serum (FBS) (HyClone) and 100 units/ml of penicillin and 100 μg/ml streptomycin. The cells were incubated at 37°C in a humidified incubator in the presence of 5% CO_2_. To passage, A549 and HeLa cells, the cells were washed with Dulbecco's phosphate-buffered saline (HyClone) and detached with 1× Trypsin-EDTA (0.025% trypsin and 0.01% EDTA) (Invitrogen).

### Plaque formation assay

The plaque assay was performed in A549 and HeLa cells as previously described [41]. The cells were seeded at 1.8 × 10^5^ cells into a 24-well tissue culture plate and incubated at 37°C with 5% CO_2_ overnight. The culture medium was removed and replaced with fresh medium supplemented with 10% FBS. The cells were infected with bacteria in triplicate at multiplicity of infection (MOI) of 5:1, 10:1 or 100:1 at 37°C with 5% CO_2_ for 2 h. Thereafter, the infected cell monolayers were washed once with PBS and maintained in culture medium containing 250 μg/ml kanamycin (Invitrogen) for 24 h to kill extracellular bacteria. The infected cells were fixed with 4% formaldehyde and stained with 1% (w/v) crystal violet for 2 min. The plaques were visualized by eye and confirmed by the observation under microscopy [41].

### Intracellular survival assays

Bacterial uptake and survival were determined in triplicate using kanamycin protection assays as previously described [48]. A549 and HeLa cells were seeded at 1.8 ×10^5^ cell per well into a 24-well plate and incubated at 37°C in 5% CO_2_ overnight. The cells were infected with *B*. *pseudomallei* at MOI of 5:1 and 10:1 for 2 h. Monolayers were washed three times with PBS and incubated with complete media containing 250 μg/ml kanamycin. To determine the surviving intracellular bacteria, the cells were washed for 3 times and lysed with 0.1% v/v Triton X-100 (Sigma) at 4-, 8- and 12-h post infection. Serial dilutions of the lysate were dropped on Columbia agar plates to enumerate bacterial colonies. The assay was performed in two independent experiments.

### MNGC formation assay

MNGC formation was performed as previously described [26]. A549 and HeLa cells were seeded at 1×10^4^ cells/well in 96-well plate and incubated for overnight at 37°C with 5% CO_2_. *B*. *pseudomallei* infection was performed in triplicate at MOI of 50:1 for 2 h. Monolayers were washed and treated with 250 μg/ml of kanamycin. The infected cells were further incubated for 10 h, then washed and fixed with 4% paraformaldehyde in PBS for 30 min. The fixed cells were washed with 50% ethanol followed by 90% ethanol for 5 min each, air dried and stained with Giemsa stain (Merck). MNGCs were examined under a light microscope and quantified using ImageJ software version 1.52n (http://rsb.info.nih.gov/ij/). An MNGC was defined as a cell having 3 or more nuclei since two nuclei within a cell may result from nuclear division without cytokinesis [16]. For each field, the total number of nuclei in MNGCs and the total number of MNGCs were determined. Percent MNGCs was calculated by number of nuclei in MNGCs x 100/total number of nuclei. Average MNGC size was calculated by total number of nuclei in MNGCs/total number of MNGCs.

### Immunostaining

Immunostaining was performed on *B*. *pseudomallei* infected cells as previously described by Srinon V et al., 2019 with some modifications [25]. Briefly, A549 cells and HeLa cell were seeded at 5 × 10^5^ cells/well on a sterile glass coverslip in a 6-well tissue culture plate and incubated for overnight at 37°C with 5% CO_2_. The monolayers were infected with *B*. *pseudomallei* at MOI of 50:1 for 2 h, after which the cells were washed and the extracellular bacteria were killed with 250 μg/ml kanamycin. The infected cells were further incubated for 8 h. The cells were washed with PBS, fixed with 4% paraformaldehyde in PBS for 30 min and permeabilised with 0.5% triton X-100 for 30 minutes. After washing three times, the permeabilised cells were incubated with 1:200 of 4B11 (2.5 μg/ml) monoclonal antibody specific to *B*. *pseudomallei* capsular polysaccharide [49] at 37°C for 1 h. Cells were then washed three times with PBS followed by incubation with goat anti-mouse IgG conjugated with Alexa Fluor 488 at dilution of 1:1,000 (Invitrogen) for *B*. *pseudomallei* detection, phalloidin conjugated with Alexa Fluor 647 at dilution of 1:1,000 (Invitrogen) for actin staining and Hoechst 33258 (1:1,000) (Invitrogen) to nuclear staining at 37°C for 1 h. Stained cells were washed three times with PBS. The cover slips were mounted on glass slides using 8 μl of ProLong Gold antifade reagent (Invitrogen). Confocal microscopy was performed with a laser scanning confocal microscope (LSM 700; Carl Zeiss) using a 100× objective lense with oil-immersion and Zen software (2010 edition, Zeiss, Germany). The excitation and emission wavelengths were 496/519 for Alexa Fluor 488, 352/461 for Hoechst 33258 and 594/633 for Alexa Fluor 647.

### Whole genome sequence analysis

Genomic DNA was extracted from 1.5 ml of overnight bacterial culture in LB broth using the QIAmp DNA mini kit (Qiagen). Library was prepared for 150-base-read with Ion Xpress Plus Fragment Library kit (Life Technologies) and next-generation sequencing was performed on Ion Torrent platform (Life Technologies). The short reads were mapped to the reference *B*. *pseudomallei* K96243 genome using CLC genomic workbench version 12.0 (CLC Bio-Qiagen). The sequence reads were deposited in the NCBI database. The accession numbers for K96243 type II and A4 are SRR11848390 and SRR11848389, respectively.

### Validation of gene deletions by PCR and DNA sequencing

The genomic loss of plaque-forming defective strains was confirmed by PCR followed by DNA sequencing. The primers were designed for a flanking region covering both edges of gene deletion. Gene loss was validated by PCR for *bimA* (*bpss1492*), *hcp5* (*bpss1498*), *clpv5* (*bpss1502*), *vgrG5* (*bpss1503*), *bpss1509*, *bopA* (*bpss1524*), *bopE* (*bpss1525*) and *bipD* (*bpss1529*). These genes were located in the genomic deletion region in chromosome 2 and predicted to be involved in invasion, intracellular replication and cell-to-cell spreading of bacteria [20, 22, 24, 27, 50–52].

### Statistical analysis

Student’s unpaired t-test was used to compare means of different groups or conditions. The analysis was performed using GraphPad Prism 6 (Graph Pad Software, Inc.). *P* value ≤ 0.05 was considered statistically significant.

## Results

### Detection of *B*. *pseudomallei* defective in plaque formation

To determine whether *B*. *pseudomallei* isolates of different origins were able to induce plaques in host cells, we infected A549 and HeLa cell lines with 67 isolates of *B*. *pseudomallei* at MOI of 5:1 and examined plaque formation. These isolates included 52 clinical isolates, 11 environmental isolates, 2 isolates of isogenic morphotypes II and III of K96243, and 2 isolates of isogenic types II and III which were generated from strain 153. The source and details of these isolates are described in Materials and Methods and shown in Table 1 and S1 Table. The fifty clinical isolates were obtained from 49 patients who were admitted with melioidosis at Nakhon Phanom Hospital. Of these, one patient with recurrent infection was enrolled in our cohort, from whom primary and relapse isolates were analysed. The patients’ age ranged from 23 to 77 years, median = 54 years and interquartile range (IQR) = 44–63 years. Thirty-one patients (63.3%) were male. Eighteen patients (36.7%) died within one year of follow-up (range 0–77 days (median = 8 days and IQR = 3–20 days) after enrollment. The clinical specimens with positive cultures from these patients included blood (N = 37), pus (N = 8), sputum (N = 3), urine (N = 1) and bullae fluid (N = 1) (S1 Table).

**Table 1:** Plaque formation of clinical and environmental isolates of *B*. *pseudomallei* in A549 and HeLa cells.

Source (Strain)	No. of isolates	Number of isolates with plaque formation	
		HeLa cell	A549 cell
Clinical isolates			
Nakhon Phanom	50	50	50
Khon Kaen (K96243)	1	1	1
Ubon Ratchathani (153)	1	1	1
Environmental isolates			
Ubon Ratchathani	11	10	10
Laboratory isolates	4	3	3
Total	67	65	65

As shown in Table 1, plaque formation in both A549 and HeLa cells were observed for all 52 clinical isolates. There was no plaque formation in one environmental isolate (A4) and one isogenic morphotype isolate (K96243 type II). We next evaluated plaque formation for each of these isolates at higher MOI of 10:1 and 100:1, however no plaques were observed following K96253 type II and A4 infection of either A549 or HeLa cells.

### Plaque-forming-defective *B*. *pseudomallei* isolates are incapable of intracellular replication

**Fig 1:**
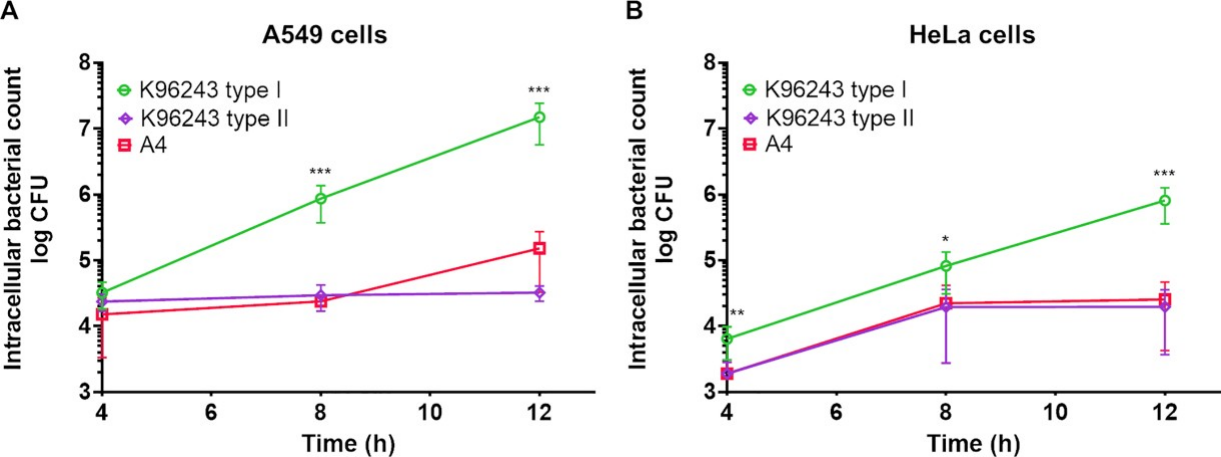
*B*. *pseudomallei* strains K96243 type II and A4 are defective in intracellular replication in A549 and HeLa cells. K96243 type I and plaque-forming-defective strains K96243 type II and A4 were used to infect A549 cells (A) at a multiplicity of infection (MOI) of 5 or HeLa cells (B) at MOI of 10. Number intracellular bacteria at 4-, 8- and 12-h post infection was determined. The assays were performed in triplicate in two independent experiments. The number of bacteria was counted by drop plate technique and the data represent means ± standard deviation. *P ≤ 0.01; ** *P* ≤ 0.005; *** *P* ≤ 0.001.

Intracellular counts of plaque-forming-defective *B*. *pseudomallei* isolates were quantified at 4, 8 and 12 h after infection. The plaque-forming-defective isolates, *B*. *pseudomallei* K96243 type II and A4 survived in both A549 cells and HeLa cells after infection (Fig 1A and 1B). However, the numbers of intracellular bacteria of both isolates did not increase at 12 h post infection compared with K96243 type I (*P* < 0.001 for all comparisons).

### Growth rates of plaque-forming-defective isolates are comparable to K96243 type I

The incapability of intracellular replication of K96242 type II and A4 led us to suspect that the plaque-defective strains might have growth rate impairment. We thus determined growth rate by performing growth curve analyses of *B*. *pseudomallei* in enrichment medium. Our results showed that the growth rate of *B*. *pseudomallei* K96243 type II and A4 were comparable to K96243 type I (S1 Fig). At log phase, the doubling times of K96243 type I, K96243 type II and A4 were 37.71 min, 35.57, min and 37.58 min, respectively when they were grown in LB at 37°C with shaking at 200 rpm. The results indicated that K96243 type II and A4 normally replicate in media but are unable to replicate within host cells.

### Plaque-forming-defective *B*. *pseudomallei* isolates do not induce actin-tails in epithelial cells

**Fig 2:**
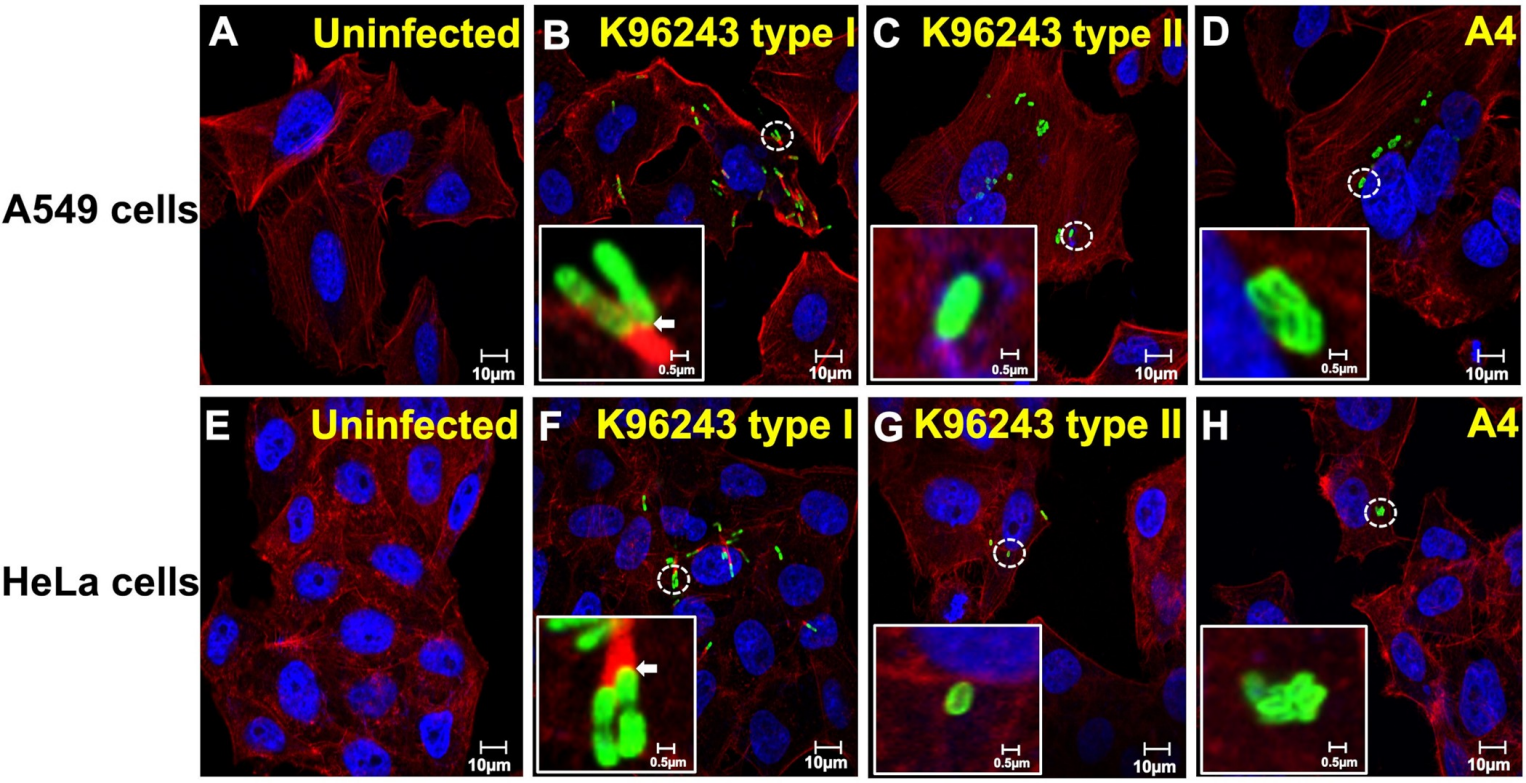
*B*. *pseudomallei* K96243 type II and A4 do not induce actin-tail formation in A549 and HeLa cells. A549 cells (A) and HeLa cells (E) were infected with *B*. *pseudomallei* K96243 type I (B and F), K96243 type II (C and G) and A4 (D and H) at MOI of 50. Immunofluorescence staining was performed at 8 h post-infection. The cells were stained with monoclonal antibody 4B11 specific to *B*. *pseudomallei* capsular polysaccharide to indicate bacteria in green, phalloidin to indicate F-actin in red and Hoechst 33258 to indicate host DNA in blue. The co-localization between bacteria and actin is shown in yellow (arrows). Scale Bars, 0.5 and 10 μm.

Actin-based motility is essential for intracellular and intercellular movement of *B*. *pseudomallei* and subsequent plaque formation. Immunofluorescence staining was performed to determine actin-tail formation in A549 and HeLa cells infected with K96243 type I, K96243 type II and A4 (Fig 2). In comparison to uninfected cells (Fig 2A and 2E), the cells infected with *B*. *pseudomallei* K96243 type I showed numerous intracellular bacteria with actin polymerization at rear poles of the bacteria and the evidence of bacterial movement inside the cells and to nearby cells (Fig 2B and 2F). In contrast, the cells infected with K96243 type II and A4 showed no actin-tail formation which indicated no bacterial motility inside the cells. Instead, there were a small number of bacteria accumulated in the infected cells (Fig 2C, 2D, 2G and 2H). The results indicated that the non-plaque forming isolates are incompetent in actin-tail induction and movement.

### Plaque-forming-defective *B*. *pseudomallei* isolates induce less MNGC formation

**Fig 3:**
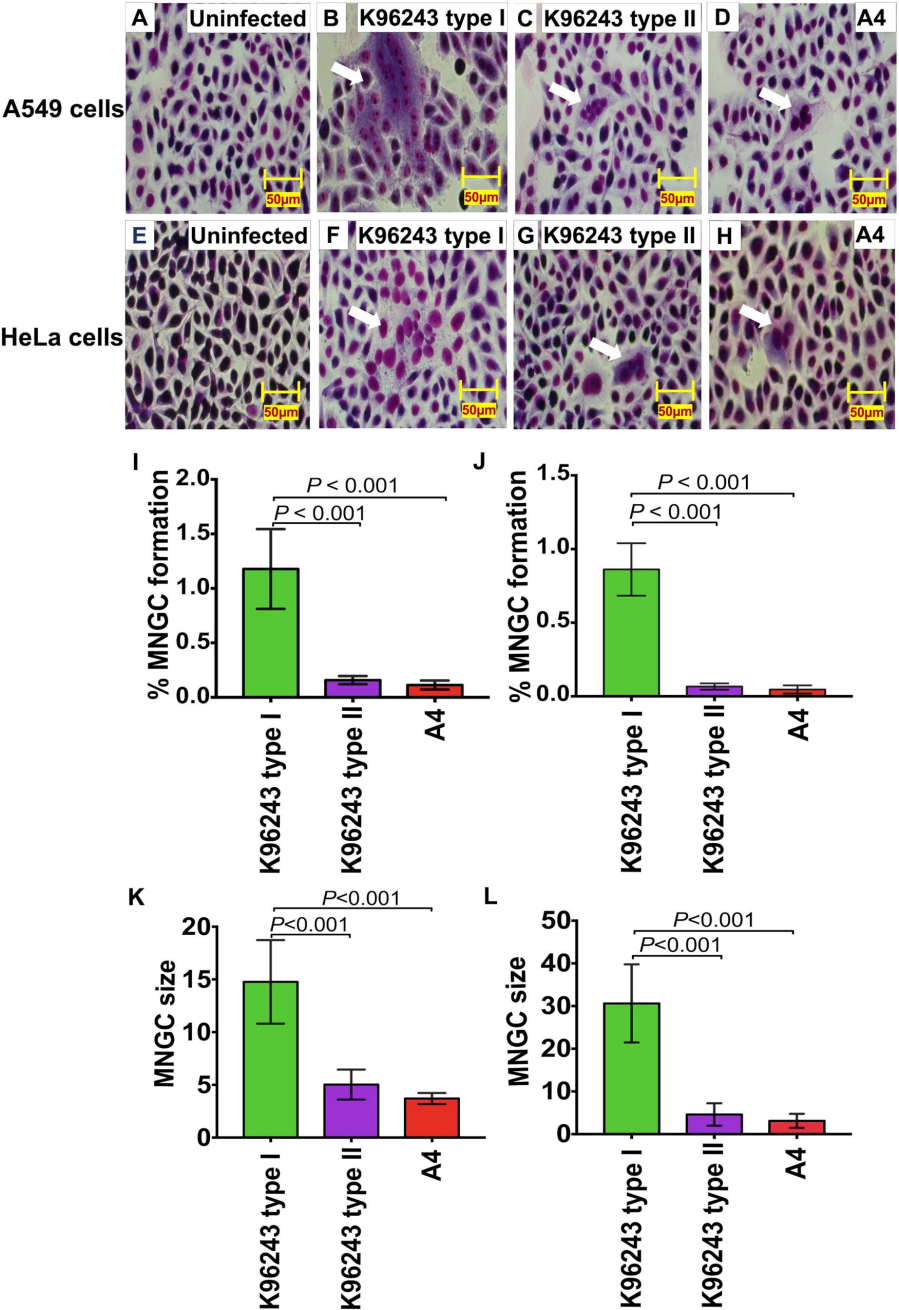
*B*. *pseudomallei* K96243 type II and A4 are defective in MNGC formation in A549 and HeLa cells. A549 cells (A) and HeLa cells (E) were infected with *B*. *pseudomallei* K96243 type I (B and F) and K96243 type II (C and G) and A4 (D and H) at MOI of 50 at 37°C for 12 h. The cells were stained with Giemsa stain. Percent MNGC formation in A549 cells (I) and HeLa cells (J) were quantified by number of nuclei in MNGCs x 100/total number of nuclei. The average size of MNGC in A549 (K) and HeLa cells (L) are shown as means ± standard deviation. Average MNGC size was calculated by total number of nuclei in MNGCs/total number of MNGCs. All assays were performed on two independent experiments in triplicate. Student’s t-test was performed to test the differences between bacterial strains. Scale Bars, 50 μm.

A notable feature of *B*. *pseudomallei* is the ability to induce the formation of MNGC; plaque formation represents MNGC death [20]. We thus determined the difference in MNGC formation in A549 and HeLa cells infected with K96243 type I, K96243 type II and A4 (Fig 3). In comparison to uninfected cells (Fig 3A and 3E), all *B*. *pseudomallei* isolates were able to form MNGC in both epithelial cell lines (Fig 3B–3D and 3F–3H). However, we found that the percentage of MNGC formation by *B*. *pseudomallei* K96243 type II and A4 was significantly lower than that of *B*. *pseudomallei* K96243 type I (*P* < 0.001 for all comparisons, Fig 3I and 3J). Furthermore, we measured the MNGC size in infected cells and found that the average MNGC size of plaque-forming-defective isolates was significantly smaller than those of control K96243 type I (*P* < 0.001 for all comparisons, Fig 3K and 3L).

### Whole genome sequencing revealed large genomic deletion in chromosome 2 of plaque-forming-defective *B*. *pseudomallei* isolates

**Fig 4:**
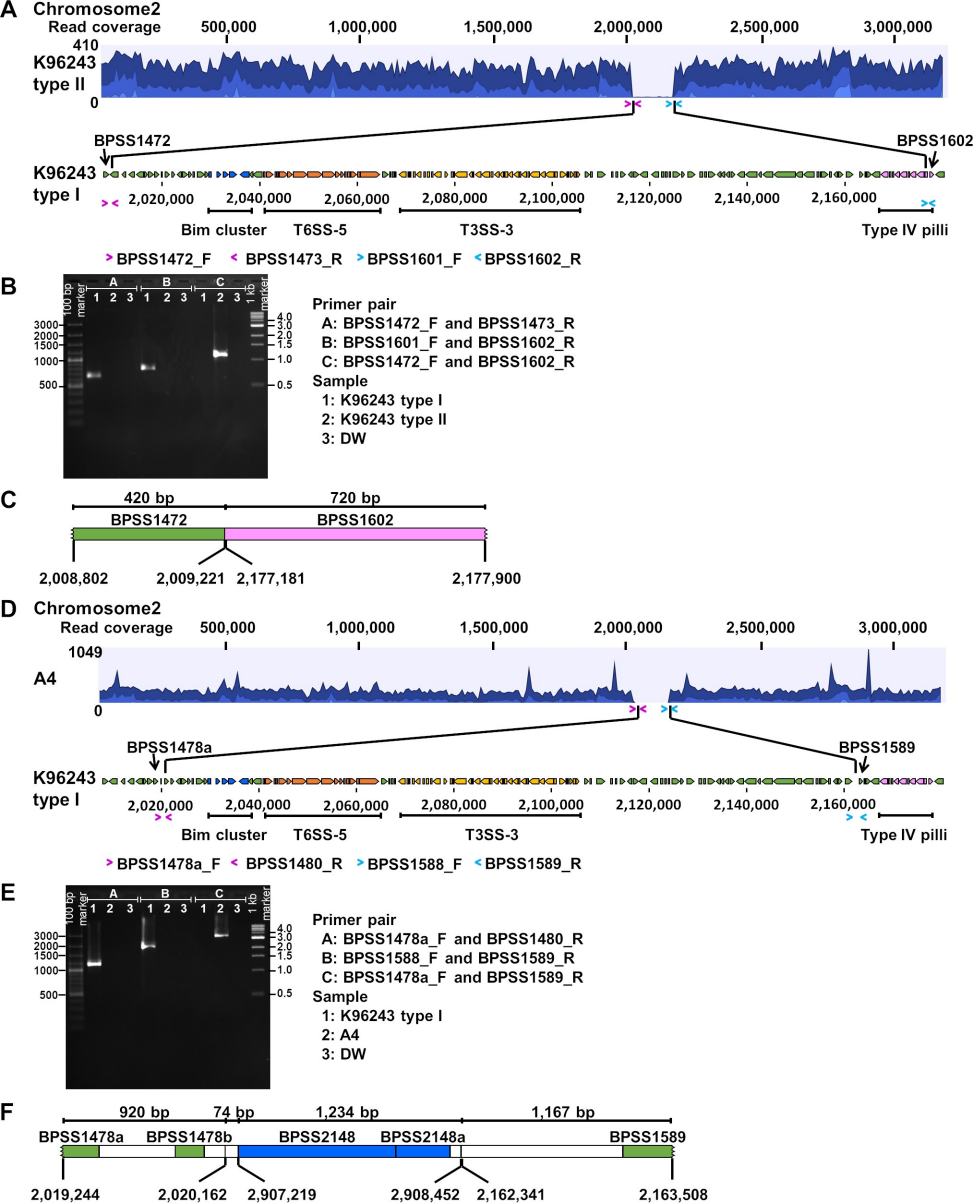
Large genomic loss in the chromosome 2 of *B*. *pseudomallei* K96243 type II and A4. Analyses of K96243 type II and A4 are shown in A-C and D-F respectively. Whole genome sequencing was performed on the Ion Torrent platform. Short reads from K96243 type II and A4 genomes were mapped against the reference genome of *B*. *pseudomallei* K96243 type I (A and D). B and E show PCR amplification of K96243 type II and A4 chromosomal DNA for left edge of the deletion (group A), right edge of the deletion (group B) and the flanking region (group C). Lane 1 is K96243 type I. Lane 2 is K96243 type II (B) or A4 (E). Lane3 is DW. C and F show gene arrangement of flanking region of the deletion in the chromosome 2 of K96243 type II and A4, respectively.

To investigate the potential genes responsible for the defects in bacterial invasion, MNGC formation and plaque-forming characteristics, we examined the genome of *B*. *pseudomallei* K96243 type II and A4 (Fig 4). With short reads mapping to the reference genome K96243, MLST of K96243 type II was identified as ST10, the same ST as the parental isolate, K96243 type I. Nineteen non-synonymous SNPs were found in 7 genes from chromosomes 1 and 2 of K96243 type II including *bpsl0500*, *bpsl1559*, *bpsl2010*, *bpsl2353*, *bpss1194* (10 SNPs), *bpss1195* (4 SNPs) and *bpss1197*. While no genomic loss was found in chromosome 1, the genomic data identified a read mapping missing for approximately 170-kb deletion between *bpss1472* and *bpss1602* in chromosome 2 of K96243 type II (Fig 4A). The region of genomic loss was verified by PCR and sequencing with primer pairs designed to amplify 1,140-bp flanking the deletion region of K96243 type II (Fig 4A and 4B). The sequencing of PCR products of K96243 type II identified a 167,959 bp deletion in chromosome 2 at position 2,009,222–2,177,180 (Fig 4C). The 130 deleted genes (*bpss1473* –*bpss1601*) are listed in S2 Table.

Resequencing of short reads from strain A4 to reference genome K96243 demonstrated that MLST of A4 was ST185. We found 4,912 non-synonymous SNPs in chromosomes 1 and 2 of A4. Only 5 genomic islands (GI1, 7, 10, 14 and 16) were present in A4 compared with 16 GIs of K96243. In addition, a 150-kb genomic loss between *bpss1478b* and *bpss1589* in chromosome 2 was observed. The deleted genes are listed in S2 Table. This region overlapped the deletion region in K96243 type II (Fig 4D). Primer pairs to amplify 3,395-bp flanking the deletion region were designed and then PCR was performed to confirm genomic loss of A4 (Fig 4D and 4E). The sequencing of PCR products of A4 identified a 142,178 bp deletion at position 2,020,163–2,162,340 (*bpss1480*- *bpss1588*). Moreover, we observed a rearrangement of *bpss2148* –*bpss2148a* (position 2,907,219–2,908,452) in the middle of amplicon of A4 (Fig 4F).

The common deletion region between the two isolates involved 110 genes which included Bim cluster (*bpss1489* –*bpss1493*), T6SS-5 (*bpss1496*—*bpss1513*) and T3SS-3 (*bpss1516* –*bpss1554*) (S2 Table). Previous studies have demonstrated that several genes in these clusters are important virulence factors that play roles in intracellular survival and MNGC formation [16, 20–30].

### PCR analyses confirmed the deletion of genes involved in host cell interaction in plaque-forming-defective isolates

Intracellular survival and MNGC formation of *B*. *pseudomallei* have been reported to be responsible by genes in the deletion region [20, 22, 24, 27, 50–52]. We confirmed whether *bimA* (*bpss1492*), *hcp5* (*bpss1498*), *clpv5* (*bpss1502*), *vgrG5* (*bpss1503*), *bpss1509*, *bopA* (*bpss1524*), *bopE* (*bpss1525*) and *bipD* (*bpss1529*) genes were deleted in plaque-forming-defective isolates by PCR. Primer sequences are shown in S3 Table. PCR results of *bimA*, *hcp5*, *clpv5*, *vgrG5*, *bpss1509*, *bopA*, *bopE* and *bipD* for K96243 type I control demonstrated products of 122, 173, 136, 152, 196, 156, 393 and 201 bp, respectively (S2 Fig). In contrast, PCR failed to detect products of all target genes from the genomic DNA of *B*. *pseudomallei* K96243 type II and A4 isolates. The data suggest a defective mechanism of bacterial-host cell interaction that was dependent on these genes in the plaque-forming-defective strains.

## Discussion

Plaque formation is the final scenario of infection *in vitro* that represents the virulence of intracellular *B*. *pseudomallei* [40, 42]. This study demonstrated that *B*. *pseudomallei* isolates are distinct in plaque-forming efficiency. All isolates from clinical samples induced high plaque-forming efficiency while only one isolate (strain A4) from the environment and one isogenic morphotype isolate (K96243 type II) were identified as plaque-forming-defective isolates. The two defective isolates grew well in enrichment broth but showed attenuation in intracellular replication, actin polymerization and MNGC formation in A549 and HeLa cells. Our genome analyses further indicated that both isolates had massive gene loss at the same region although they were distinct genotypes.

The identification of isolates with defects in plaque-formation associated with genomic loss has not been reported in *B*. *pseudomallei*. In this study, we found 110 deleted genes in the 150-kb deletion region in nature for environmental isolate strain A4. The region included major virulence systems such as Bim cluster, T3SS-3 and T6SS-5 systems. It is known that Bim cluster has a key function in actin polymerization which is required for intracellular movement of *B*. *pseudomallei* [24, 25] and T6SS-5 plays a major role for cell-to-cell fusion and intracellular spread [27, 29, 30]. T3SS-3 is essential for bacterial escape from endocytic vesicles [20–23]. Our findings emphasize that this deletion region is crucial for the intracellular lifestyle and inter-cellular spreading of *B*. *pseudomallei* during infection. Our finding of the same 170-kb deletion region with 130 deleted genes in isogenic morphotype K96243 type II generated under nutritional starvation from parental type I suggests that the deletion may be associated with adaptation and evolution of *B*. *pseudomallei*. This genetic event can be a result of genetic recombination and is likely to be a common characteristic of *B*. *pseudomallei* rather than of the specific genotypes.

Our data also demonstrated that the plaque-forming-defective isolate from the environment (strain A4) can infect but was impaired in replication in host cells. This would suggest that this environmental isolate is unlikely to cause severe infection. The deletion region included major virulence genes known to be crucial for the intracellular lifestyle of *B*. *pseudomallei* infection; mutants defective in one or more of these genes have been shown to be attenuated for virulence in animal models [25, 27]. Although these genes are required for virulence, they may not be essential for *B*. *pseudomallei* to survive in the environment.

Although the plaque-forming–defective isolates were not found in clinical specimens, genomic deletions associated with antibiotic resistance, adaptation and other functions have been reported in clinical isolates. *B*. *pseudomallei* isolates with large genomic loss containing penicillin-binding protein 3 (PBP3) have been isolated from six Thai patients during prolonged exposure to ceftazidime treatment [53]. A natural >130 kb genomic deletion has been reported in a *B*. *pseudomallei* gentamicin-susceptible strain. The deletion region includes the *amrAB-oprA* operon which encodes an efflux pump [54]. In addition, Price et al reported an evidence of a 245-kb deletion in chromosome 2 of 37-month persistent strain, MSHR1655 and a 285-kb genomic loss containing 221 genes in a variant of *B*. *pseudomallei* isolate MSHR6686 arising from 139-month persistent infection in an Australian patient [55]. Hayden et al reported a decay of 330-kb in chromosome 2 (*bpss1250*—*bpss1482)* of a persistent strain 1258b [56]. Recently, Pearson et al showed evidence of genomic deletions during within-host evolution of *B*. *pseudomallei* isolates from a human case where melioidosis severity lessened with time. The shift towards avirulence was demonstrated by clinical data and testing in a mouse model. The deletions included genes related to O-antigen, capsular polysaccharide, motility, and T3SS. We mapped the deletion regions of strains K96243 type II and A4 in our study to *B*. *pseudomallei* MSHR1435 genome as the reference, and found the deletions located in region 6 associated with T3SS and T6SS in their study [57]. Furthermore, a large chromosomal deletion has also been discovered in an environmental isolate, *B*. *pseudomallei* RF80, a ST507 strain from Thailand. This strain was known to be negative to a species-specific TTS1 PCR assay for *B*. *pseudomallei* [58]. The strain was selected for inclusion on the inclusivity panel of *B*. *pseudomallei* strains tested for DNA-based assay development [59]. A further analysis by us using its genome to compare with the genome of K96243 has shown that it had lost approximately 273 genes starting from *bpss1331* to *bpss1603* in chromosome 2. This gene loss region included all the virulence genes described in both K96243 type II and A4 genomes, as well as the missing of T3SS-1 (*bpss1390* –*bpss1408*) genes.

Genomic loss may be a common process of in *B*. *pseudomallei* evolution for fitness with different environments. Genomic decay is often observed in the evolution from free living to obligate intracellular bacteria e.g., *Mycobacterium leprae* and *Burkholderia mallei* [60, 61]. Studies showed the occurrence of several point mutations and gene deletions of *Pseudomonas aeruginosa* and *B*. *pseudomallei* representing within-host adaptation from chronic infections and during treatment [55, 56, 62]. This mechanism may benefit bacteria entering a dormant state within the host [55, 56]. This adaptive mechanism has also been reported in laboratory conditions for other bacteria. For examples, the growth of numerous generations in laboratory conditions can induce gene loss in *Escherichia coli* and *Salmonella* spp. [63, 64].

## Conclusions

Our plaque-forming assay and whole genome analyses identified 1/11 of environmental isolates (9%) and 1/4 isogenic morphotype isolates (25%) of *B*. *pseudomallei* from Thailand as plaque-forming-defective. We showed evidence of large genomic loss of many genes related to the functions of intracellular replication and cell-to-cell spreading in these isolates. A limitation of this study is that the number of environmental isolates for our analysis was small and we did not observe how the genomic loss occurred. However, the proportion and distribution of plaque-forming-defective *B*. *pseudomallei* isolates across regions and countries remain unknown. It is possible that *B*. *pseudomallei* is more diverse in virulence in nature and the presence of the regions encoding Bim cluster, T3SS-3 and T6SS-5 genes correlate with the number of melioidosis cases. Further epidemiological and phenotypic studies as well as animal testing are required to identify the risk of human infection and the virulence of plaque-defective strains.

## Supporting information

S1 Table Source of *B*. *pseudomallei* isolates and patient information. (DOCX)

S2 Table List of deleted genes in chromosome 2 of K96243 type II and A4. (DOCX)

S3 Table Primer sequences for PCR validation of genomic loss. (DOCX)

S1 Fig Growth curves of *B*. *pseudomallei* strains K96243 type I, K96243 type II and A4. All strains show similar growth patterns in LB medium. The error bars represent standard errors.
(TIF)

S2 Fig Confirmation of genomic deletion in *B*. *pseudomallei* K96243 and A4. The loss of several genes in deletion region including Bim cluster, T6SS-5 and T3SS-3 were verified by conventional PCR. The amplification from genomic DNA of K96243 type I (Lane1), K96243 type II (Lane2) and A4 (Lane3) were performed for detection of *bimA*, *hcp5*, *clpV5*, *vgrG5*, *bpss1509*, *bopA*, *bopE* and *bipD*.
(TIF)
