# Supplementary material for: Genomic loss in environmental and isogenic morphotype isolates of Burkholderia pseudomallei is associated with intracellular survival and plaque-forming efficiency
Source: PLoS Negl Trop Dis. 2020 Sep 29;14(9):e0008590. doi: 10.1371/journal.pntd.0008590 (PMC7546507; doi:10.1371/journal.pntd.0008590)
Supplement: S1 Table — (DOCX) [file pntd.0008590.s001.docx]

**Table S1.** Source of *B. pseudomallei* isolates and patient information

| **Subject** | **Sex** | **Age** | **Outcome at 28-day of enrollment** | **Outcome after enrollment for 1 year** | **Strain** | **Specimen** |
| --- | --- | --- | --- | --- | --- | --- |
| 40-002 | Female | 23 | Survived | Survived | DR40002A | Pus |
| 40-003 | Female | 54 | Died | Died | DR40003A | Sputum |
| 40-004 | Male | 69 | Survived | Survived | DR40004A | Pus |
| 40-005 | Male | 34 | Died | Died | DR40005A | Blood |
| 40-006 | Male | 50 | Survived | Unable to contact | DR40006A | Blood |
| 40-007 | Male | 36 | Survived | Survived | DR40007A | Blood |
| 40-008 | Male | 67 | Survived | Survived | DR40008A | Pus |
| 40-009 | Male | 72 | Survived | Survived | DR40009A | Blood |
| 40-010 | Male | 66 | Survived | Survived | DR40010A | Blood |
| 40-011 | Female | 61 | Survived | Survived | DR40011A | Sputum |
| 40-012 | Male | 43 | Survived | Survived | DR40012A | Blood |
| 40-013 | Male | 65 | Survived | Survived | DR40013A | Blood |
| 40-014 | Male | 77 | Died | Died | DR40014A | Blood |
| 40-015 | Male | 75 | Died | Died | DR40015A | Blood |
| 40-016 | Male | 35 | Survived | Survived | DR40016A | Blood |
| 40-017 | Female | 71 | Survived | Survived | DR40017A | Blood |
| 40-020 | Female | 45 | Died | Died | DR40010A | Blood |
| 40-022* | Female | 48 | Survived | Survived | DR40022A | Blood |
|  |  |  |  | Survived | DR40022E | Pus |
| 40-023 | Male | 72 | Survived | Survived | DR40023A | Blood |
| 40-025 | Male | 49 | Survived | Survived | DR40025A | Blood |
| 40-027 | Male | 48 | Survived | Died | DR40027A | Pus |
| 40-028 | Female | 50 | Survived | Survived | DR40028A | Blood |
| 40-031 | Male | 47 | Survived | Survived | DR40031A | Blood |
| 40-032 | Male | 55 | Died | Died | DR40032A | Blood |
| 40-033 | Male | 61 | Died | Died | DR40033A | Blood |
| 40-035 | Male | 73 | Died | Died | DR40035A | Sputum |
| 40-036 | Male | 64 | Survived | Survived | DR40036A | Blood |
| 40-037 | Male | 60 | Died | Died | DR40037A | Blood |
| 40-040 | Female | 52 | Died | Died | DR40040A | Pus |
| 40-041 | Male | 55 | Survived | Survived | DR40041A | Blood |
| 40-042 | Female | 60 | Died | Died | DR40042A | Blood |
| 40-043 | Male | 36 | Survived | Survived | DR40043A | Pus |
| 40-044 | Female | 39 | Survived | Survived | DR40044A | Bullae fluid |
| 40-047 | Male | 42 | Died | Died | DR40047A | Blood |
| 40-048 | Female | 60 | Died | Died | DR40048A | Urine |
| 40-049 | Male | 61 | Survived | Died | DR40049A | Blood |
| 40-050 | Male | 50 | Survived | Survived | DR40050A | Blood |
| 40-051 | Female | 49 | Survived | Survived | DR40051A | Blood |
| 40-052 | Male | 40 | Survived | Survived | DR40052A | Blood |
| 40-053 | Female | 40 | Survived | Survived | DR40053A | Blood |
| 40-054 | Female | 44 | Survived | Survived | DR40054A | Blood |
| 40-055 | Female | 53 | Survived | Survived | DR40055A | Blood |
| 40-056 | Male | 62 | Survived | Survived | DR40056A | Blood |
| 40-057 | Female | 70 | Survived | Survived | DR40057A | Pus |
| 40-058 | Female | 58 | Died | Died | DR40058A | Blood |
| 40-059 | Male | 58 | Survived | Survived | DR40059A | Blood |
| 40-060 | Male | 31 | Survived | Survived | DR40060A | Blood |
| 40-067 | Male | 63 | Died | Died | DR40067A | Blood |
| 40-073 | Female | 44 | Died | Died | DR40073A | Blood |
| * Recurrence melioidosis within 1 year after enrollment | | | | | | |
