# Supplementary material for: Genomic loss in environmental and isogenic morphotype isolates of Burkholderia pseudomallei is associated with intracellular survival and plaque-forming efficiency
Source: PLoS Negl Trop Dis. 2020 Sep 29;14(9):e0008590. doi: 10.1371/journal.pntd.0008590 (PMC7546507; doi:10.1371/journal.pntd.0008590)
Supplement: S2 Table — (DOCX) [file pntd.0008590.s002.docx]

**Table S2. List of deleted genes in chromosome 2 of K96243 type II and A4**

| **Locus tag** | **Symbol** | **Description** | **K96243 type II** | **A4** |
| --- | --- | --- | --- | --- |
| BPSS1473 |  | hypothetical protein | Absent | Present |
| BPSS1474 |  | DeoR family transcriptional regulator | Absent | Present |
| BPSS1475 |  | transmembrane sugar transporter | Absent | Present |
| BPSS1476 |  | mannitol dehydrogenase | Absent | Present |
| BPSS1477 |  | GntR family transcriptional regulator | Absent | Present |
| BPSS1478 | *rspA* | mandelate racemase | Absent | Present |
| BPSS1478a | *rspB* | dehydrogenase | Absent | Present |
| BPSS1478b |  | pseudogene | Absent | Present |
| BPSS1480 |  | hypothetical protein | Absent | Absent |
| BPSS1481 |  | hypothetical protein | Absent | Absent |
| BPSS1482 | *nadE* | NAD synthetase | Absent | Absent |
| BPSS1483 |  | TetR family transcriptional regulator | Absent | Absent |
| BPSS1484 |  | hypothetical protein | Absent | Absent |
| BPSS1485 |  | hypothetical protein | Absent | Absent |
| BPSS1486 |  | hypothetical protein | Absent | Absent |
| BPSS1487 |  | anaerobically induced outer membrane protein | Absent | Absent |
| BPSS1488 |  | hypothetical protein | Absent | Absent |
| BPSS1489 | *bimB* | hypothetical protein | Absent | Absent |
| BPSS1490 | *bimC* | N-acetylmuramoyl-L-alanine amidase | Absent | Absent |
| BPSS1491 | *bimA* | hypothetical protein | Absent | Absent |
| BPSS1492 | *bimD* | hypothetical protein | Absent | Absent |
| BPSS1493 | *bimE* | hypothetical protein | Absent | Absent |
| BPSS1494 | *virG* | two-component system response regulator | Absent | Absent |
| BPSS1495 | *virA* | two-component system sensor kinase | Absent | Absent |
| BPSS1496 | *tssA* | hypothetical protein | Absent | Absent |
| BPSS1497 | *tssB* | hypothetical protein | Absent | Absent |
| BPSS1498 | *hcp* | hypothetical protein | Absent | Absent |
| BPSS1499 | *tssC* | hypothetical protein | Absent | Absent |
| BPSS1500 | *tssD* | hypothetical protein | Absent | Absent |
| BPSS1501 | *tssE* | hypothetical protein | Absent | Absent |
| BPSS1502 | *clpV* | Clp-type ATPase chaperone protein | Absent | Absent |
| BPSS1503 | *vgrG* | hypothetical protein | Absent | Absent |
| BPSS1504 | *tssF* | hypothetical protein | Absent | Absent |
| BPSS1505 | *tssG* | hypothetical protein | Absent | Absent |
| BPSS1506 | *tssH* | hypothetical protein | Absent | Absent |
| BPSS1507 | *tssI* | hypothetical protein | Absent | Absent |
| BPSS1508 | *tssJ* | hypothetical protein | Absent | Absent |
| BPSS1509 | *tssK* | hypothetical protein | Absent | Absent |
| BPSS1510 | *tssL* | hypothetical protein | Absent | Absent |
| BPSS1511 | *icmF* | hypothetical protein | Absent | Absent |
| BPSS1512 | *tssM* | hypothetical protein | Absent | Absent |
| BPSS1513 | *tssN* | hypothetical protein | Absent | Absent |
| BPSS1514 | *folE* | GTP cyclohydrolase I | Absent | Absent |

**Table S2 (continued). List of deleted genes in chromosome 2 of K96243 type II and A4**

| **Locus tag** | **Symbol** | **Description** | **K96243 type II** | **A4** |
| --- | --- | --- | --- | --- |
| BPSS1515 |  | pseudogene | Absent | Absent |
| BPSS1516 | *bopC* | effector of type III secretion system | Absent | Absent |
| BPSS1517 |  | hypothetical protein | Absent | Absent |
| BPSS1518 |  | transposase | Absent | Absent |
| BPSS1519 |  | transposase | Absent | Absent |
| BPSS1520 | *bprC* | AraC family transcriptional regulator | Absent | Absent |
| BPSS1521 | *bprD* | effector of type III secretion system | Absent | Absent |
| BPSS1522 | *bprB* | two-component system response regulator | Absent | Absent |
| BPSS1523 | *bicP* | chaperone | Absent | Absent |
| BPSS1524 | *bopA* | intercellular spread protein | Absent | Absent |
| BPSS1525 | *bopE* | G-nucleotide exchange factor | Absent | Absent |
| BPSS1526 | *bapC* | invasion protein | Absent | Absent |
| BPSS1527 | *bapB* | acyl carrier protein | Absent | Absent |
| BPSS1528 | *bapA* | effector of type III secretion system | Absent | Absent |
| BPSS1529 | *bipD* | translocator of type III secretin system | Absent | Absent |
| BPSS1530 | *bprA* | HNS-like regulatory protein | Absent | Absent |
| BPSS1531 | *bipC* | translocator of type III secretin system | Absent | Absent |
| BPSS1532 | *bipB* | translocator of type III secretin system | Absent | Absent |
| BPSS1533 | *bicA* | chaperone protein SicA | Absent | Absent |
| BPSS1534 | *bsaZ* | structural protein of type III secretion system protein SpaS | Absent | Absent |
| BPSS1535 | *bsaY* | structural protein of type III secretion system protein | Absent | Absent |
| BPSS1536 | *bsaX* | structural protein of type III secretion system protein | Absent | Absent |
| BPSS1537 | *bsaW* | structural protein of type III secretion system protein SpaP | Absent | Absent |
| BPSS1538 | *bsaV* | structural protein of type III secretion system protein | Absent | Absent |
| BPSS1539 | *bsaU* | structural protein of type III secretion system protein | Absent | Absent |
| BPSS1540 | *bsaT* | structural protein of type III secretion system protein | Absent | Absent |
| BPSS1541 | *bsaS* | ATP synthase SpaL | Absent | Absent |
| BPSS1542 | *bsaR* | chaperone protein | Absent | Absent |
| BPSS1543 | *bsaQ* | structural protein of type III secretion system protein | Absent | Absent |
| BPSS1544 | *bsaP* | structural protein of type III secretion system protein | Absent | Absent |
| BPSS1545 | *bsaO* | structural protein of type III secretion system protein | Absent | Absent |
| BPSS1546 | *bsaN* | AraC family transcriptional regulator | Absent | Absent |
| BPSS1547 | *bsaM* | structural protein of type III secretion system protein | Absent | Absent |
| BPSS1548 | *bsaL* | structural protein of type III secretion system protein | Absent | Absent |
| BPSS1549 | *bsaK* | structural protein of type III secretion system protein | Absent | Absent |
| BPSS1550 | *bsaJ* | structural protein of type III secretion system protein | Absent | Absent |
| BPSS1551 | *orgA* | structural protein of type III secretion system protein | Absent | Absent |
| BPSS1552 | *orgB* | structural protein of type III secretion system protein | Absent | Absent |
| BPSS1553 | *bprP* | Regulator of type III secretion system | Absent | Absent |
| BPSS1554 | *bprQ* | Regulator of type III secretion system | Absent | Absent |
| BPSS1555 |  | peptidase | Absent | Absent |
| BPSS1556 |  | MarR family transcriptional regulator | Absent | Absent |
| BPSS1557 |  | glyoxalase | Absent | Absent |

**Table S2 (continued). List of deleted genes in chromosome 2 of K96243 type II and A4**

| **Locus tag** | **Symbol** | **Description** | **K96243 type II** | **A4** |
| --- | --- | --- | --- | --- |
| BPSS1558 | *kgtP* | alpha-ketoglutarate permease | Absent | Absent |
| BPSS1559 |  | LysR family transcriptional regulator | Absent | Absent |
| BPSS1560 | *mdlB* | L(+)-mandelate dehydrogenase | Absent | Absent |
| BPSS1561 |  | carboxypeptidase | Absent | Absent |
| BPSS1562 |  | kumamolisin | Absent | Absent |
| BPSS1563 |  | LysE family translocator | Absent | Absent |
| BPSS1564 |  | transcriptional regulator AsnC | Absent | Absent |
| BPSS1565 |  | hypothetical protein | Absent | Absent |
| BPSS1566 |  | phosphate transporter | Absent | Absent |
| BPSS1567 |  | hypothetical protein | Absent | Absent |
| BPSS1568 |  | acyl-CoA ligase (AMP-forming), exosortase A system-associated | Absent | Absent |
| BPSS1569 |  | N-acylhomoserine lactone-dependent regulatory protein | Absent | Absent |
| BPSS1570 |  | N-acylhomoserine lactone synthase | Absent | Absent |
| BPSS1571 |  | NADH oxidoreductase | Absent | Absent |
| BPSS1572 | *tauC* | taurine ABC transporter permease | Absent | Absent |
| BPSS1573 | *tauB* | taurine ABC transporter ATP-binding protein | Absent | Absent |
| BPSS1574 | *tauA* | taurine ABC transporter substrate-binding protein | Absent | Absent |
| BPSS1575 | *tauD* | taurine dioxygenase | Absent | Absent |
| BPSS1576 |  | hypothetical protein | Absent | Absent |
| BPSS1577 | *bcsA* | cellulose synthase catalytic subunit | Absent | Absent |
| BPSS1578 |  | hypothetical protein | Absent | Absent |
| BPSS1578a |  | hypothetical protein | Absent | Absent |
| BPSS1579 |  | hypothetical protein | Absent | Absent |
| BPSS1580 |  | cellulose biosynthesis protein | Absent | Absent |
| BPSS1581 | *bcsZ* | endo-1,4-D-glucanase | Absent | Absent |
| BPSS1582 | *bcsB* | cellulose synthase regulator | Absent | Absent |
| BPSS1582a |  | hypothetical protein | Absent | Absent |
| BPSS1583 | *hipB* | transcriptional regulator | Absent | Absent |
| BPSS1584 | *hipA* | regulatory protein | Absent | Absent |
| BPSS1585 |  | hypothetical protein | Absent | Absent |
| BPSS1586 |  | LysR family transcriptional regulator | Absent | Absent |
| BPSS1587 | *speB* | agmatinase | Absent | Absent |
| BPSS1588 |  | hypothetical protein | Absent | Absent |
| BPSS1589 |  | hypothetical protein | Absent | Present |
| BPSS1590 |  | hypothetical protein | Absent | Present |
| BPSS1591 |  | hypothetical protein | Absent | Present |
| BPSS1592 |  | type III secretion system protein | Absent | Present |
| BPSS1593 | *pilV* | type IV pilus biosynthesis protein | Absent | Present |
| BPSS1594 |  | hypothetical protein | Absent | Present |
| BPSS1595 | *pilS* | major pilin subunit | Absent | Present |
| BPSS1596 | *pilR* | type IV pilus biosynthesis protein | Absent | Present |
| BPSS1597 | *pilQ* | type IV pilus biosynthesis protein | Absent | Present |
| BPSS1598 |  | hypothetical protein | Absent | Present |

**Table S2 (continued). List of deleted genes in chromosome 2 of K96243 type II and A4**

| **Locus tag** | **Symbol** | **Description** | **K96243 type II** | **A4** |
| --- | --- | --- | --- | --- |
| BPSS1599 | *pilO* | type IV pilus biosynthesis protein | Absent | Present |
| BPSS1600 | *pilN* | type IV pilus biosynthesis protein | Absent | Present |
| BPSS1601 |  | type IV pilus biosynthesis protein | Absent | Present |
