# Supplementary material for: Genomic loss in environmental and isogenic morphotype isolates of Burkholderia pseudomallei is associated with intracellular survival and plaque-forming efficiency
Source: PLoS Negl Trop Dis. 2020 Sep 29;14(9):e0008590. doi: 10.1371/journal.pntd.0008590 (PMC7546507; doi:10.1371/journal.pntd.0008590)
Supplement: S3 Table — (DOCX) [file pntd.0008590.s003.docx]

**Table S3.** Primer sequences for PCR validation of genomic loss

| **Primer name** | **Sequence (5' -> 3')** | **References** |
| --- | --- | --- |
| *bpss1472*_F | GTGCTTGCACACCCTCTTTG | This study |
| *bpss1473*_R | CGAATTCCTGCGGCTTTACG | This study |
| *bpss1601*_F | GCAATCGGTTGCAAAAAGCG | This study |
| *bpss1602*_R | GAGGATGATGCCCTTCGTGT | This study |
| *bpss1478a*_F | GAAGGAATTGACGCTGTGCG | This study |
| *bpss1480*_R | CTGGATTTCGAGGACGATGC | This study |
| *bpss1588*_F | AGAGAACATCACGCACCTCG | This study |
| *bpss1589*_R | ATCCACGCATTCCACAGCAG | This study |
| *bpss1492*_F | CATCCGAAACGCCGTCAATC | This study |
| *bpss1492*_R | CGTCGCCCGGATTTTTCTTC | This study |
| *bpss1498*_F | GTGCTGATCACCCACATGGA | This study |
| *bpss1498*_R | CAGCCATTCGTCCAGTTTGC | This study |
| *bpss1502*_F | CACCACGTGGAGCGAATACA | This study |
| *bpss1502*_R | CTGATAGCGCTCCTTCAGCC | This study |
| *bpss1503*_F | TTCCACACGAAGGACGAAGG | This study |
| *bpss1503*_R | TCGAAATCCTGGCGGTAGTG | This study |
| *bpss1509*_F | GCTGCAACCGCAACATTTTC | This study |
| *bpss1509*_R | GCCCGGATATTCGACGTAGC | This study |
| *bopA* F | GTATTTCGGTCGTGGGAATG | Pumirat et al., 2010 |
| *bopA* R | GCGATCGAAATGCTCCTTAC | Pumirat et al., 2010 |
| *bopE* F | CGGCAAGTCTACGAAGCGA | Pumirat et al., 2010 |
| *bopE* R | GCGGCGGTATGTGGCTTCG | Pumirat et al., 2010 |
| *bipD* F | GGACTACATCTCGGCCAAAG | Pumirat et al., 2010 |
| *bipD* R | ATCAGCTTGTCCGGATTGAT | Pumirat et al., 2010 |
