# Supplementary figures and images for: Genomic loss in environmental and isogenic morphotype isolates of Burkholderia pseudomallei is associated with intracellular survival and plaque-forming efficiency
Source: PLoS Negl Trop Dis. 2020 Sep 29;14(9):e0008590. doi: 10.1371/journal.pntd.0008590 (PMC7546507; doi:10.1371/journal.pntd.0008590)

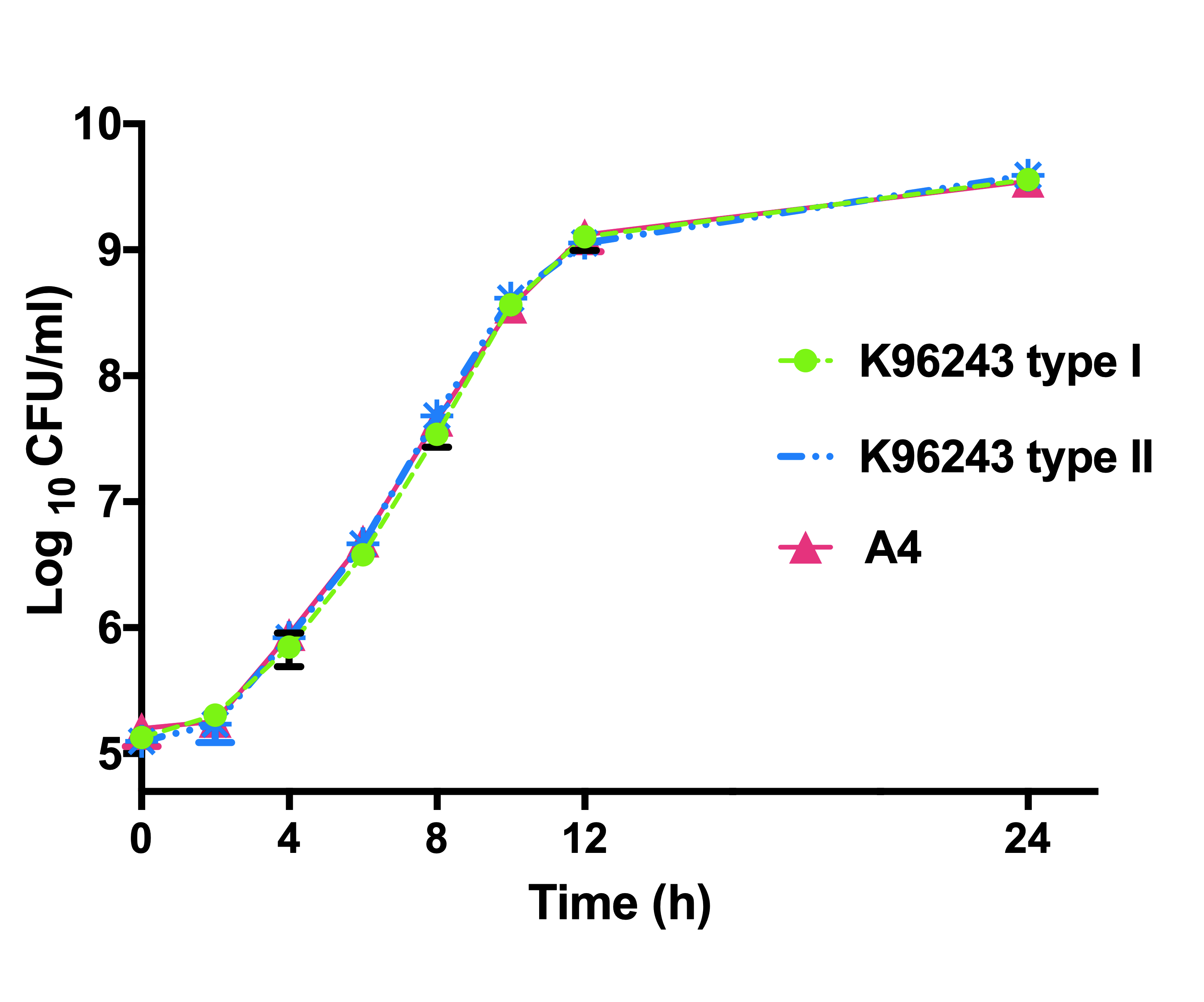

Supplement: S1 Fig — All strains show similar growth patterns in LB medium. The error bars represent standard errors. (TIF) [file pntd.0008590.s004.tif]

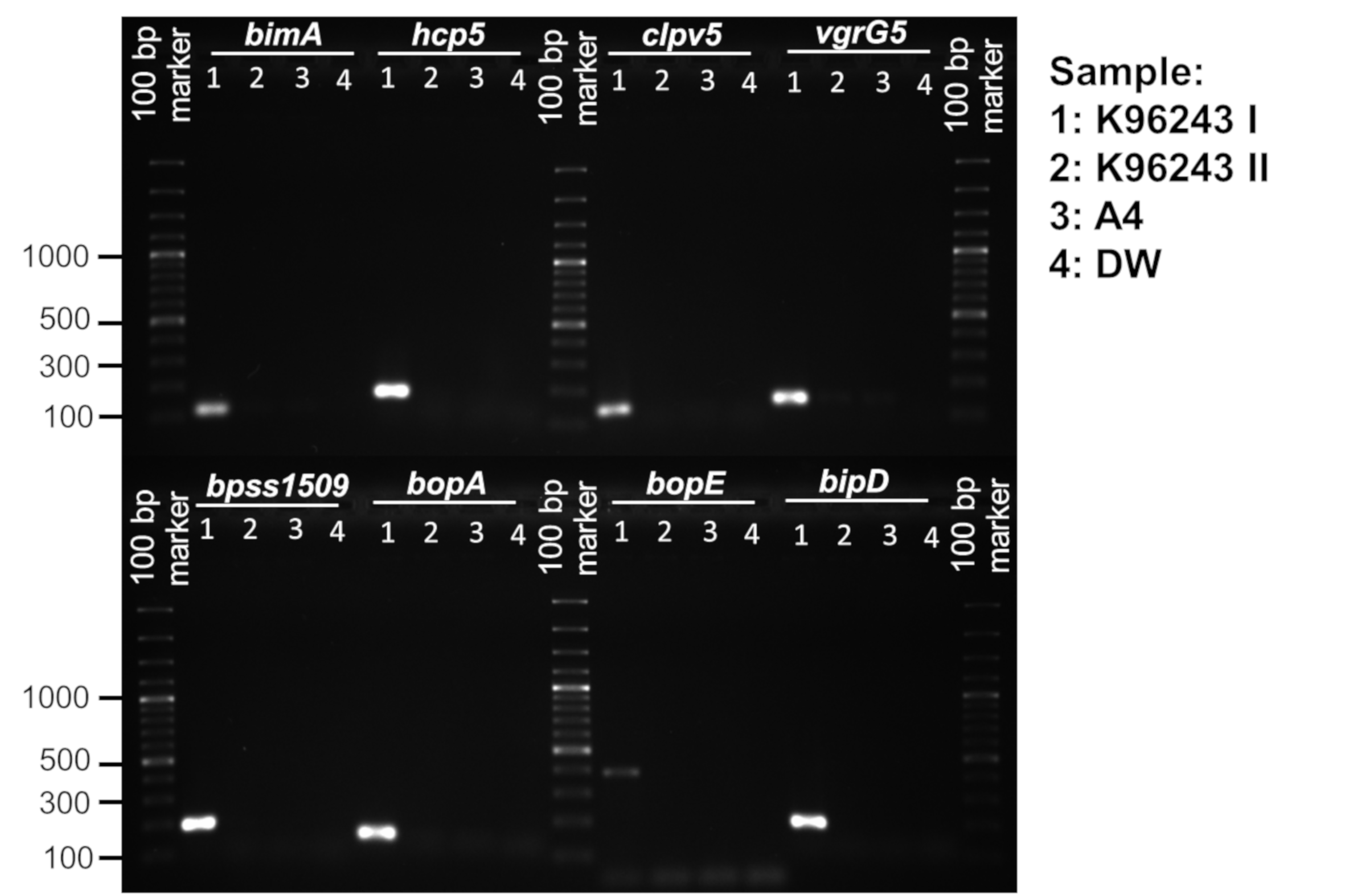

Supplement: S2 Fig — The loss of several genes in deletion region including Bim cluster, T6SS-5 and T3SS-3 were verified by conventional PCR. The amplification from genomic DNA of K96243 type I (Lane1), K96243 type II (Lane2) and A4 (Lane3) were performed for detection of bimA, hcp5, clpV5, vgrG5, bpss1509, bopA, bopE and bipD. (TIF) [file pntd.0008590.s005.tif]
